# Bibliometrics analysis on the research status and trends of adult-onset Still’s disease: 1921-2021

**DOI:** 10.3389/fimmu.2022.950641

**Published:** 2022-07-18

**Authors:** Aining Qin, Jing Sun, Chao Gao, Chunying Li

**Affiliations:** ^1^ School of Nursing, Peking University, Beijing, China; ^2^ Department of Community Nursing, School of Nursing, Peking University, Beijing, China; ^3^ Department of Rheumatology and Immunology, Peking University People’s Hospital, Beijing, China; ^4^ Information and Reference Department, Peking University Health Science Library, Beijing, China

**Keywords:** Still’s disease, adult-onset, scopus, bibliometrics [MeSH], trends, epidemiology

## Abstract

**Objectives:**

The aim of this research is to discuss the research status, hotspots, frontiers and development trends in the field of adult-onset Still’s disease (AOSD) based on bibliometrics and visual analysis by CiteSpace software.

**Methods:**

The relevant research articles on AOSD from 1921 to 2021 were retrieved from the Scopus database. CiteSpace software was used to form a visual knowledge map and conduct analysis for the countries/regions, journals, authors, keywords, clusters, research hotspots and frontiers of the included articles.

**Results:**

There were 2,373 articles included, and the number of articles published during 1921-2021 is increasing. The country with the highest number of articles published was Japan (355, 14.96%), followed by the United States (329, 13.86%) and France (215, 9.06%). The author with the highest number of publications is Ansell, Barbara M. (30, 1.26%), and the author with the highest co-citation frequency is Yamaguchi, Masaya (703). *Clinical Rheumatology* is the journal with the highest publication frequency. The top five cluster groups were “joint”, “differential diagnosis”, “prednisolone”, “methotrexate” and “macrophage activation syndrome”. The diagnosis, treatment and pathogenesis of AOSD form the main research fields, and prognosis and complications are the research hotspots and trends.

**Conclusions:**

The global research field in AOSD has expanded in the past 100 years. The complications and new pathogenesis of AOSD are hotspots in this field and need further study in the future.

## Introduction

Adult-onset Still’s disease (AOSD) is a kind of systemic inflammatory disease with unknown etiology that can accumulate in various systems of the whole body, with a global incidence rate of (0.16–0.40)/100,000 and an estimated prevalence rate of (1-34)/1,000,000 (1). The incidence rate of males in Japan is 0.22/100,000 and that of females is 0.34/100,000 (2). Due to the low prevalence of AOSD, there is still a lack of extensive and reliable epidemiological data in clinics at the present stage (3). A study on Poland revealed that the incidence of AOSD in urban areas of Poland (0.33/100,000) was significantly higher than that in rural areas (0.29/100,000) (4). The age distribution of the AOSD is bimodal, with one peak occurring between 15-25 years and the other between 36-45 years (5). The typical clinical manifestations of AOSD mainly include fever (60-100%), arthritis or arthralgia (70-100%) and maculopapular red rash (60-80%) (6). Atypical clinical manifestations such as sore throat or pharyngitis, myalgia, lymphadenopathy, and hepatosplenomegaly may also occur in some patients (7). In addition, the prevalence of mental disorders in AOSD patients is increased due to the long-term chronic course of the disease, which especially affects their emotions (8). In general, the prognosis of AOSD is good (9), but there is still a mortality rate of 3% (3, 10), and some serious complications can also lead to death (11), such as macrophage activation syndrome (MAS) (12–14%) (12), thrombotic thrombocytopenic purpura (<1%) (13), disseminated intravascular coagulation disease (14), and acute respiratory distress syndrome (15).

Many factors, including genetic factors (16), infection (17), and immune dysfunction (18) may be possible causative factors of AOSD. Several studies have shown that *Human leucocyte antigen* is closely related to the occurrence of AOSD (19, 20), and the *Macrophage migration inhibitory factor gene* may increase susceptibility to the disease (21). Bacterial, viral, or parasitic infections may also cause AOSD (22). At present, the pathogenesis is still controversial, and pathophysiology studies are rare (23). Studies have shown that chemokines and pro-inflammatory cytokines, such as interferon (IFN)-γ, tumor necrosis factor (TNF)-α and interleukin (IL) are involved in the pathogenesis of AOSD (24). In the absence of markers for specific diagnosis and curative effect evaluation (25), AOSD is primarily diagnosed by excluding other diseases (26). For the possibility of misdiagnosis in AOSD (27), the average delay time of diagnosis is about four months (28). Yamaguchi criteria (29) have the highest diagnostic sensitivity of 92%, followed by Fautrel criteria (30) (87%) and Cush criteria (31) (80%). AOSD has been treated symptomatically mainly by using non-steroidal anti-inflammatory drugs (NSAIDs), steroids and disease-modifying anti-rheumatic drugs (DMARDs) (26). With the in-depth understanding of the pathogenesis, Biological Response Modifiers are gradually trying to be used for treatment (32). However, the effect of traditional treatment schemes is not satisfactory, with more than 80% of patients not relieved after only using NSAIDs (33) and 45% of patients developing hormone dependence after the use of steroids (34). At the same time, new treatments, such as Biological Response Modifiers still lack more effective clinical experimental data to verify (35).

In the past few years, a number of scholars have carried out basic and clinical studies on the pathogenesis and treatment of AOSD (36). However, few reports have analyzed the characteristics and development trend of the AOSD over a long period of time, which is not conducive for researchers to accurately grasp the occurrence, development rules and characteristics of the AOSD (37). With an increasing number of reports on the AOSD research, retrieving the research status quickly and efficiently in related fields has become a more realistic problem faced by researchers (38). Bibliometrics and visual analysis provide an important, feasible and systematic method for judging the importance of published literature by showing the author’s networks and academic exchanges, connections between scholars and the development in the field of knowledge (39). Using the results of the bibliometric analysis will not only help researchers understand the global research trends of AOSD and master the information sources of AOSD research but also help researchers understand the advantages and disadvantages of their research and quickly capture the research priorities, hotspots, and trends (40).

In this study, the research articles related to adult-onset Still’s disease in the Scopus database were selected and analyzed by using CiteSpace software. From the perspectives of bibliometrics and visual analysis, the research progress of AOSD is discussed, aiming to understand the research development trends and new trends of AOSD, identify the hotspots in this research field, and provide a reference and basis for better research on AOSD.

## Materials and methods

### Data source and search strategy

Scopus is a multi-disciplinary abstract index database launched by Elsevier in 2004 (http://www.scopus.com), which contains nearly 25,000 active titles from more than 7,000 publishing houses worldwide, covering 240 disciplinary fields such as engineering, agriculture and environmental science, biomedicine, social science, art and humanities (41). Scopus is currently the largest database of abstracts and citations in the world, providing a one-stop platform for researchers to obtain scientific and technological literature (42), which can provide a reliable data basis for this study.

We retrieved the Scopus database core dataset, and the search formula is as follows: TITLE-ABS-KEY (“adult onset still disease”) OR TITLE-ABS-KEY (“still disease”). The search time range was from 1921 to 2021. The last retrieval date was November 30, 2021.

### Inclusion and exclusion criteria

The periodical articles with research contents related to the theme of “adult-onset Still’s disease” were included by reading the titles, abstracts and keywords of the detected articles. Articles with incomplete research information, conference articles, degree papers, review articles, book content, and duplicate articles were excluded.

### Analyzing tools and statistical methods

CiteSpace is a web-based Java application for analyzing and visualizing co-citation networks (43). CiteSpace analyzes the research by using the information contained in the articles and predicts the future development of this field (44). The visual co-occurrence network is constructed with CiteSpace software.

In the CiteSpace software parameter setting, the time span is set to be from November 1921 to November 2021, the time slice of 1 year, the threshold item is selected as “Top N”, and the data of the top 10 high frequency nodes are selected for each time slice. “Pathfinder” is selected as the cutting connection mode to simplify the network structure and highlight important features.

## Analysis results and visualization

### Published outcomes and cited outcomes

**Figure 1:**
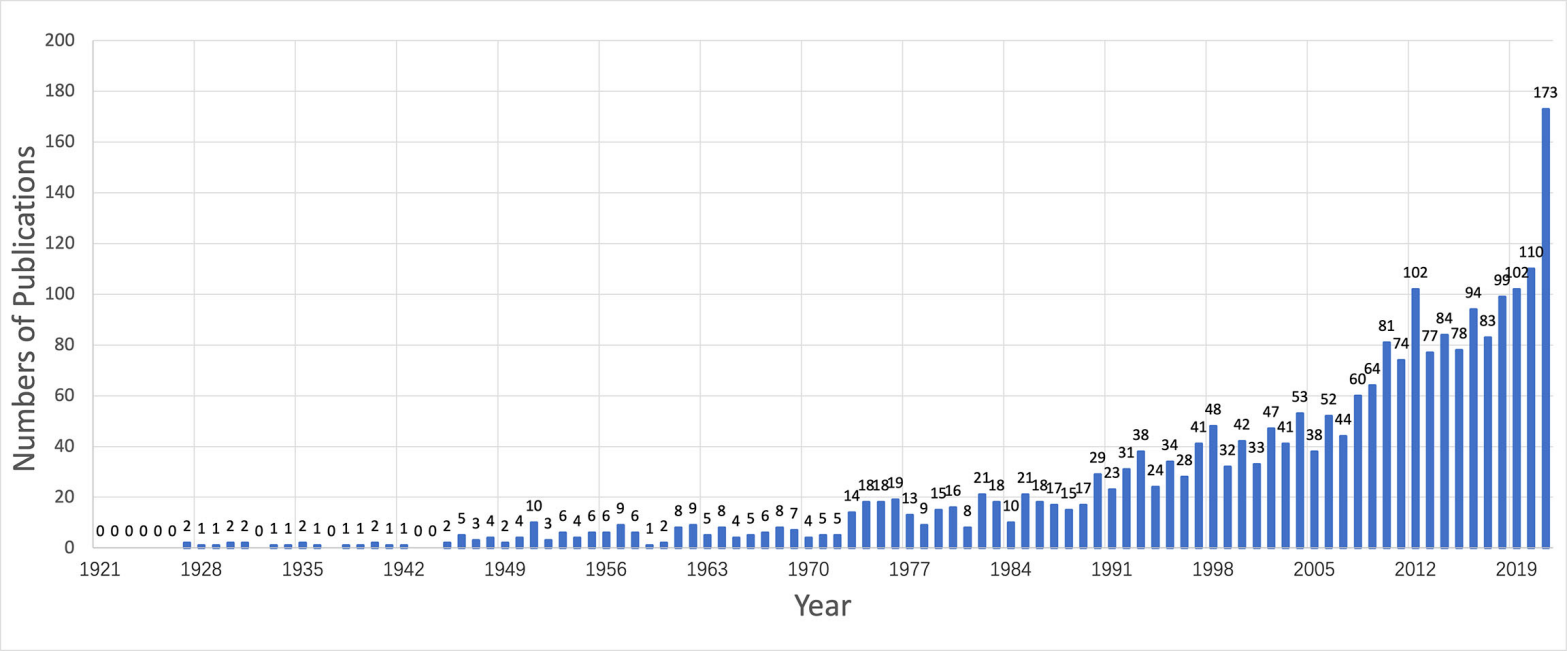
The annual quantities of adult-onset Still’s disease articles from 1921 to 2021.

A total of 2,378 articles were retrieved, and duplicated articles in the imported articles were deleted by using CiteSpace software. Finally, 2,373 articles were included. The number of articles published in the past decade has shown a steady growth trend. See **Figure 1** for details. In 2021 (173), the number of publications was approximately 2.3 times that in 2011 (74), reflecting the increased attention given by the academic community to adult-onset Still’s disease. The main research areas of AOSD are Medicine, Immunology and Microbiology, Biochemistry, Genetics and Molecular biology, accounting for 95.69% of the total frequency, and Medicine is the most frequently reported (2,291, 75.54%). See **Table 1** for details. Among the 2,373 retrieved articles, the total citation frequency was 17,968, the average citation frequency of each article was 7.57 times, and the highest citation frequency of a single article was 1,252 times. The top 10 most frequently cited articles are shown in **Table 2**.

**Table 1:** The research areas of adult-onset Still’s disease articles from 1921 to 2021.

Rank	Areas	Publications	%(N=3,033)	Rank	Areas	Publications	%(N=3,033)
1	Medicine	2,291	75.54	12	Veterinary	4	0.13
2	Immunology and Microbiology	433	14.28	13	Chemical Engineering	3	0.10
3	Biochemistry, Genetics and Molecular Biology	179	5.90	14	Environmental Science	3	0.10
4	Pharmacology, Toxicology and Pharmaceutics	29	0.96	15	Mathematics	3	0.10
5	Neuroscience	19	0.63	16	Arts and Humanities	2	0.07
6	Multidisciplinary	18	0.59	17	Computer Science	2	0.07
7	Dentistry	10	0.33	18	Engineering	2	0.07
8	Health Professions	10	0.33	19	Physics and Astronomy	2	0.07
9	Agricultural and Biological Sciences	9	0.30	20	Decision Sciences	1	0.03
10	Nursing	7	0.23	21	Earth and Planetary Sciences	1	0.03
11	Chemistry	4	0.13	22	Social Sciences	1	0.03

**Table 2:** Top 10 highly cited articles from 1921 to 2021.

Rank	Title	Total citations	Publication year	Journal	Impact factors
1	Preliminary criteria for classification of adult Still’s disease	1252	1992	Journal of Rheumatology	4.666
2	The intergroup rhabdomyosarcoma study‐I. A final report	775	1988	Cancer	6.86
3	Still’s disease in the adult.	636	1971	Annals of the Rheumatic Diseases	19.103
4	Adult Still’s disease: Manifestations, disease course, and outcome in 62 patients	547	1991	Medicine	1.889
5	Interleukin-1 receptor antagonist (anakinra) treatment in patients with systemic-onset juvenile idiopathic arthritis or adult onset Still disease: Preliminary experience in France	322	2008	Annals of the Rheumatic Diseases	19.103
6	Proposal for a new set of classification criteria for adult-onset Still disease	298	2002	Medicine	1.889
7	Rapid responses to anakinra in patients with refractory adult-onset Still’s disease	292	2005	Arthritis and Rheumatism	8.955
8	Adult‐onset Still’s disease	282	1987	Arthritis and Rheumatism	8.955
9	Remission induced by an elemental diet in small bowel Crohn’s disease	255	1987	Archives of Disease in Childhood	3.801
10	The Hyperferritinemic Syndrome: Macrophage activation syndrome, Still’s disease, septic shock and catastrophic antiphospholipid syndrome	251	2013	BMC Medicine	8.775

### Journals, authors and countries/regions distribution

These articles are published in 649 journals, with an average published volume of 3.66 articles. The journal with the most published articles is *Clinical Rheumatology*, with 80 articles, accounting for 3.37% of the total. The journals with the top 5 articles included 314 articles, accounting for 13.2% of the total, as shown in **Table 3**.

**Table 3:** Top 5 journals with the largest number of articles from 1921 to 2021.

Rank	Journal	Publications	% (N=2,373)	Impact factors
1	Clinical Rheumatology	80	3.37	2.98
2	Journal Of Rheumatology	75	3.16	4.666
3	Annals Of The Rheumatic Diseases	66	2.78	19.103
4	Modern Rheumatology	47	1.98	3.023
5	Clinical And Experimental Rheumatology	46	1.94	4.473

**Figure 2:**
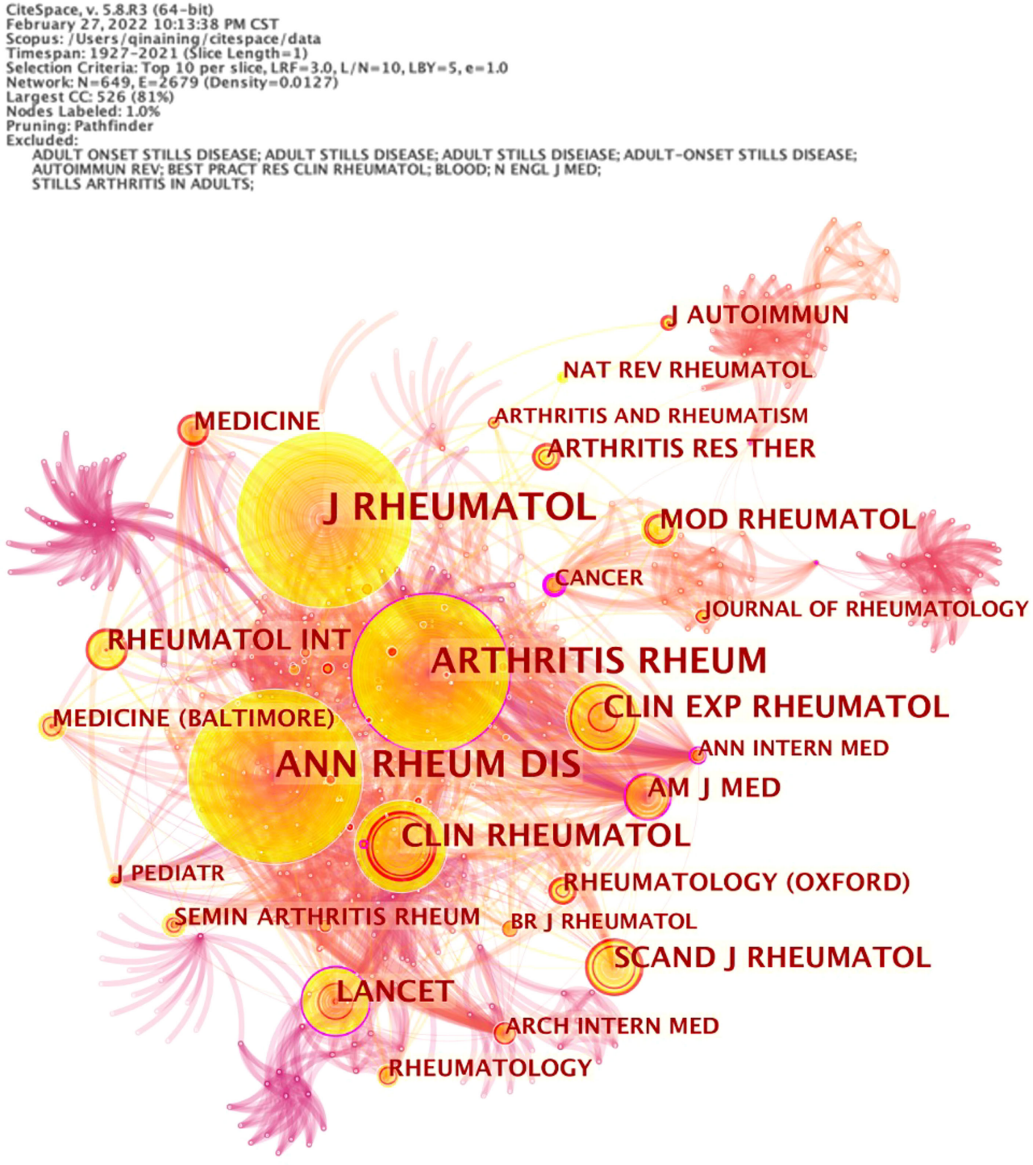
The co-citation network of adult-onset Still’s disease articles from 1921 to 2021.

**Figure 2** is the co-citation network of journals, in which the number of nodes is 649 and the number of links is 2,679. The top 5 cited journals are *Journal of Rheumatology* (1,081, 40.4%), *Annals of The Rheumatic Diseases* (893, 33.3%), *Arthritis Rheumatism* (687, 25.6%), *Clinical Rheumatology* (316, 11.8%), and *Clinical and Experimental Rheumatology* (184, 6.9%). Centrality reflects the importance of nodes, which is shown as a purple circle in the figure. The higher the centrality is, the more important the node is. The top 3 journals in centrality are *Arthritis Rheumatism* (0.10), *Journal of Rheumatology* (0.04), and *Annals of The Rheumatic Diseases* (0.03).

A total of 4,308 authors are involved in the publication of articles related to adult-onset Still’s disease. Three authors write more than 20 articles, and among them, Ansell, Barbara M. from Clinica Pediatrica ranks first with 30 articles. See **Table 4** for details.

**Table 4:** Top 10 authors by number of published works from 1921 to 2021.

Rank	Author	Publications	Citing Articles	Institution
1	Ansell, Barbara M.	30	610	Clinica Pediatrica
2	Kim, Hyoun Ah	21	276	Ajou University School of Medicine
3	Chen, Der Yuan	21	422	China Medical University College of Medicine
4	Wang, Zhihong	19	48	Shanghai Jiao Tong University School of Medicine
5	Yang, Chengde	18	133	Shanghai Jiao Tong University School of Medicine
6	Shi, Hui	18	143	Shanghai Jiao Tong University School of Medicine
7	Teng, Jialin	16	102	Shanghai Jiao Tong University School of Medicine
8	Suh, Chang Hee	16	379	Ajou University School of Medicine
9	Sun, Yue	16	111	Shanghai Jiao Tong University School of Medicine
10	Ye, Junna	15	102	Shanghai Jiao Tong University School of Medicine

**Figure 3:**
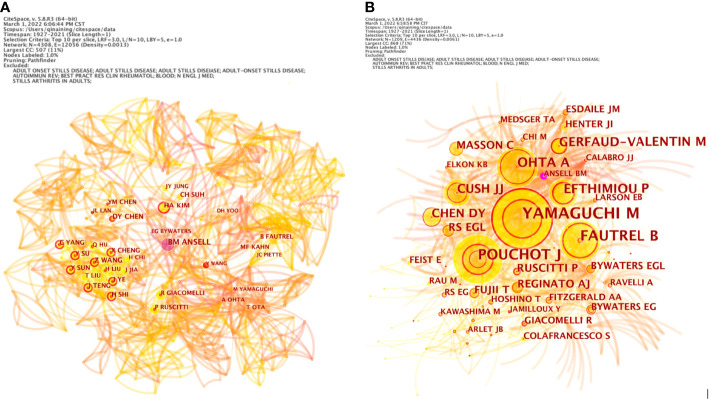
The network map of cooperation between authors **(A)** and co-authorship **(B)** of adult-onset Still’s disease articles from 1921 to 2021.

In the network map of cooperation between authors, the number of nodes is 4,308, and the number of links is 12,056, in which one node represents an author and the size of the circle represents the number of published articles by the author. The larger the node diameter is, the more published articles there are. The connection between the nodes indicates that the authors have a cooperative relationship, as shown in **Figure 3A**.

The results show that Ansell, Barbara M., Kim, Hyoun Ah, Chen, Der Yuan, Wang, Zhihong and other authors with high publication volumes each formed several independent core author groups, with close cooperation within the core author group and relatively few connections between different author groups. These authors’ research topics reflect the hot topics in the field to a certain extent, so paying attention to the research direction and content of these core author groups can better understand the development frontier and trend of adult-onset Still’s disease research.

The number of nodes in the co-authorship network is 1,229, and the number of links is 4,436. The top 5 co-authorship times ranking are Yamaguchi, Masaya (703), Pouchot, Jacques (374), Fautrel, Bruno J. (366), Ohta, Akihide (240) and Efthimiou, Petros (229), as shown in **Figure 3B**.

There are 90 countries/regions around the world paying attention to the research on adult-onset Still’s disease. Five countries have published more than 140 articles, and the top 10 countries/regions have 1,603 articles, accounting for 67.55% of the total published articles. Among them, Japan ranks first with 355 articles, accounting for 14.96% of the total number of articles, followed by the United States (329, 13.86%). See **Table 5** for details.

**Table 5:** Top 10 countries/regions with the largest number of articles from 1921 to 2021.

Rank	Country	Publications	Citing Articles	%(N=2,373)
1	Japan	355	4,091	14.96
2	United States	329	6,020	13.86
3	France	215	3,937	9.06
4	Italy	145	2,366	6.11
5	China	142	790	5.98
6	Germany	124	2,109	5.23
7	United Kingdom	99	2,580	4.17
8	South Korea	82	1,065	3.46
9	Spain	58	769	2.44
10	Türkiye	54	826	2.28

**Figure 4:**
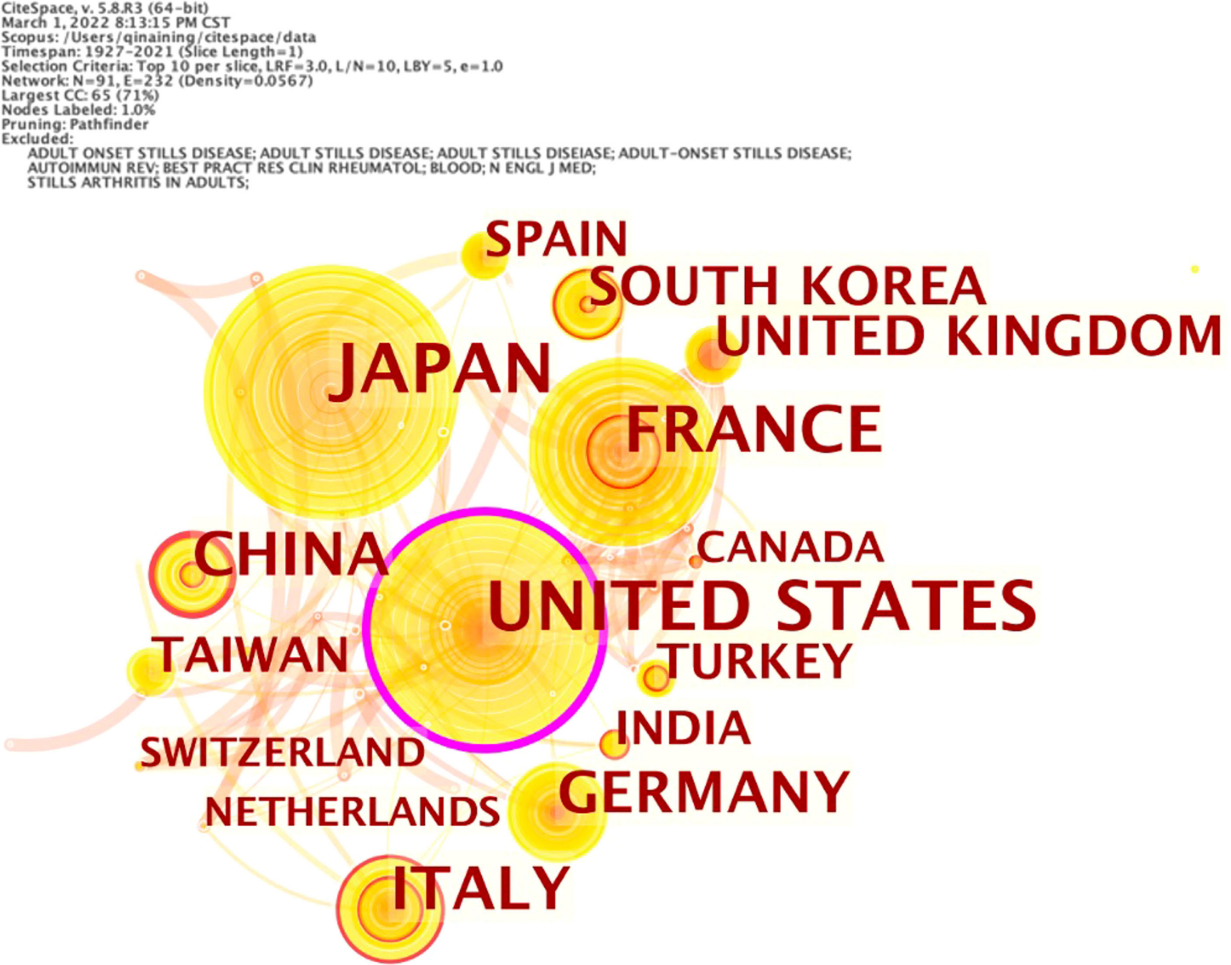
Network of cooperation among countries/regions of adult-onset Still’s disease articles from 1921 to 2021.

As shown in **Figure 4**, the number of nodes in the network of cooperation among countries/regions is 91, and the number of links is 232. The top 3 countries in the centrality ranking are the United States (0.31), France (0.06) and the United Kingdom (0.05), indicating that these 3 countries have close cooperation with other countries/regions.

### Frequency, co-occurrence analysis, cluster analysis and burst detection of keywords

**Figure 5:**
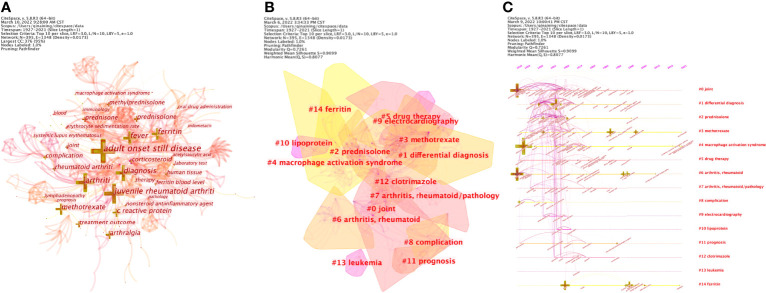
**(A)** Network of co-occurrence among keywords of adult-onset Still’s disease articles from 1921 to 2021 **(B)** Keyword clusters analysis of adult-onset Still’s disease articles from 1921 to 2021 **(C)** The timeline view of keyword clusters of adult-onset Still’s disease articles from 1921 to 2021.

Using CiteSpace software to visually analyze the keywords, we obtain the network of co-occurrence among keywords, as shown in **Figure 5A**. The number of nodes in the keyword co-occurrence network is 395, and the number of links is 1,348. As shown in **Table 6**, the high frequency keywords in the top 5 are “adult onset Still disease” (1501), “juvenile rheumatoid arthritis” (701), “fever” (663), “arthritis” (605) and “diagnosis” (437).

**Table 6:** High frequency keywords (>200) of adult-onset Still’s disease articles from 1921 to 2021.

Rank	Keyword	Frequency	Centrality
1	adult onset Still disease	1501	0.07
2	juvenile rheumatoid arthritis	701	0.10
3	fever	663	0.07
4	arthritis	605	0.08
5	diagnosis	437	0.16
6	ferritin	430	0.03
7	methotrexate	389	0.02
8	c reactive protein	291	0.00
9	arthralgia	275	0.02
10	prednisolone	219	0.02

Using the log-likelihood ratio (LLR) method in keyword clustering, a total of 28 clustering groups are obtained. Each module represents a cluster, and the larger the module is, the greater the number of keywords in the cluster. The first five cluster groups are as follows: #0 joint, #1 differential diagnosis, #2 prednisolone, #3 methotrexate, and #4 macrophage activation syndrome, as shown in **Figure 5B**. The clustering groups reflect that the research hotspots are mainly focused on the lesion location, differential diagnosis and therapeutic medication. The timeline view of keyword clusters mainly reflects the relationship between different clustering groups and the changing trend of keywords in the research process. A horizontal line represents a clustering group, and different keywords are arranged on the horizontal line in chronological order. Timeline View in CiteSpace was selected to visually analyze the keywords, and the results are shown in **Figure 5C**.

**Figure 6:**
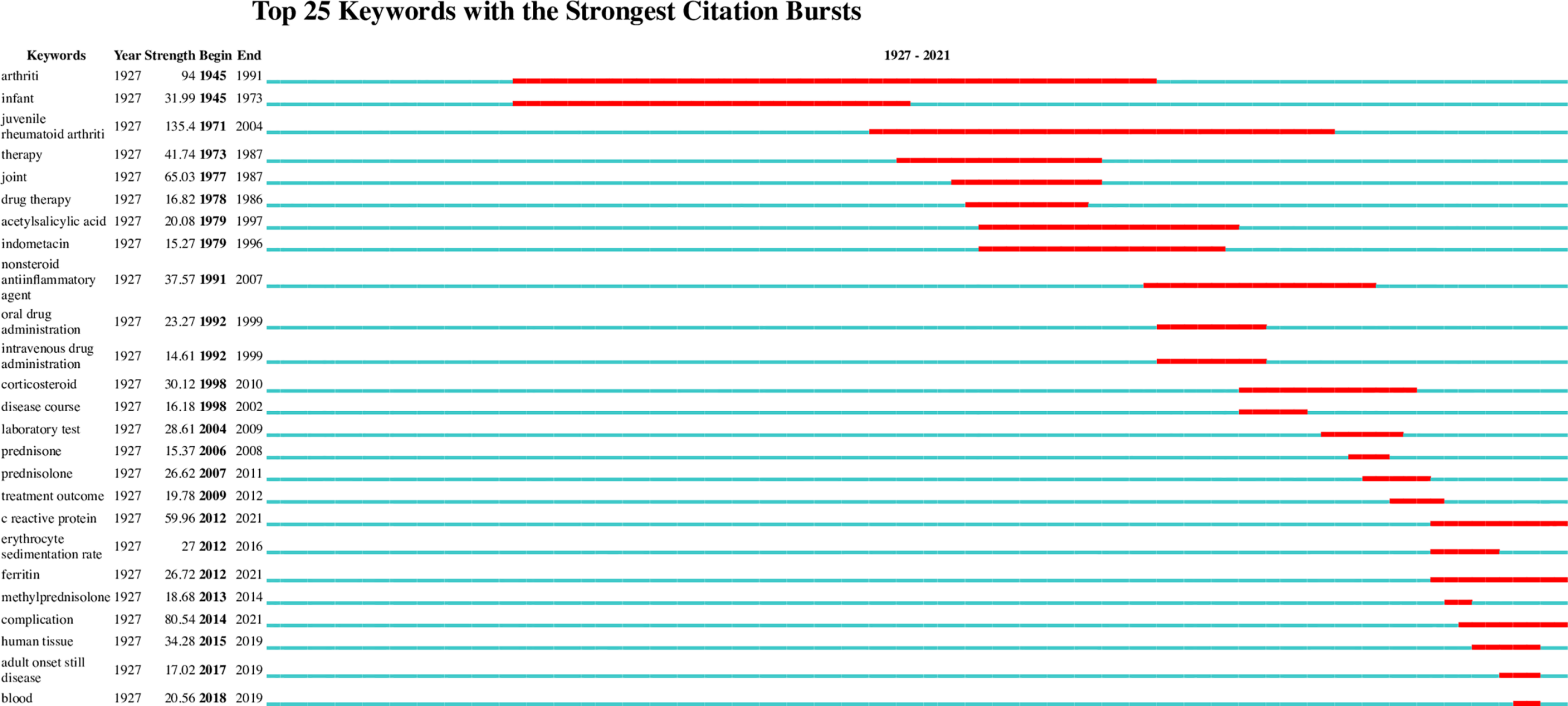
Top 25 keywords with the strongest citation bursts of adult-onset Still’s disease articles from 1921 to 2021.

Burstness refers to keywords with a sudden or significant increase in frequency in a short time (45). The larger the burst strength is, the more active the field is, and the better it can focus on research hotpots. Using the keyword burst detection function of CiteSpace software, a total of 56 burst keywords are detected, of which the top 25 are shown in **Figure 6**. Among them, “arthritis” (94) has the strongest burst strength. The keywords with strong burst strength in the past 8 years are “methylprednisolone” (2013-2014), “complication” (2014-2021), “human tissue” (2015-2019), “adult onset Still disease” (2017-2019), and “blood” (2018-2019). The change in that keyword with time can be roughly divided into three stages, in which the keywords of each stage are shown in **Table 7**.

**Table 7:** Keywords during different periods.

Time Span	Keywords
1921-1997	arthriti, infant, juvenile rheumatoid arthriti, therapy, joint, drug therapy, acetylsalicylic acid, indometacin
1998-2011	nonsteroid antiinflammatory agent, oral drug administration, corticosteroid, disease course, laboratory test, prednisolone, treatment outcome
2012-2021	c reactive protein, erythrocyte sedimentation rate, ferritin, methylprednisolone, complication, human tissue, adult onset still disease, blood

## Discussion

In this study, 2,373 articles related to AOSD from the Scopus database were analyzed by bibliometric methods. From 1921 to 2021, the number of published articles showed an overall upward trend (**Figure 1**), which can be divided into two stages. Before 1972, it was in the initial research period of AOSD, with a few articles published and a slow growth rate of literature. The basic research in this period laid a solid theoretical foundation for the development of AOSD. Since 1972, the number of articles published has increased rapidly, and there is no obvious trend of slowing down, which indicates that the research has not yet entered the mature stage, and the research of AOSD still has great space and potential. For countries, Japan, the United States and France publish the largest number of articles (**Table 5**), and the United States, France and the United Kingdom engage in relatively more international cooperation (**Figure 4**), which is basically consistent with the results of other studies (46). This may be because the improvement of gross domestic product and rapidly expanding economies result in more research funding support, thereby increasing scientific productivity (47). In addition, because developed countries have advanced medical levels and abundant scientific research resources, cooperation with developed countries can promote the improvement of scientific productivity. The results show that in scientifically weaker regions, internal cooperation will be more efficient than international cooperation (48). Therefore, we suggest that developed countries strengthen cooperation with wider countries/regions and strengthen internal cooperation and contact in relatively underdeveloped areas to promote the improvement of global standards at the AOSD medical level.

*Clinical Rheumatology* is the journal with the largest number of published articles, and *Journal of Rheumatology* is the journal with the most citations (**Table 3** and **Figure 1**). The impact factors of the two journals are above 2, indicating that the quality of AOSD-related research papers is high and has certain academic value. The article *Preliminary criteria for classification of adult Still’s disease* published by Yamaguchi, Masaya in *Journal of Rheumatology* in 1992 has the most citations (**Table 2**). This article describes a preliminary diagnostic criteria of AOSD (29), namely Yamaguchi criteria, which are now widely used.

The most representative and influential authors in the field of AOSD research have formed their own core author groups with little cooperation among them (**Figure 3**). Ansell, Barbara M.’s group focuses on the pathophysiological changes of AOSD (49, 50). Their study showed that IgG antiglobulin factor levels are elevated in AOSD patients (51). In the FII haemagglutination test for serum antigammaglobulin factors of AOSD patients, the mean FII tube titre increased, and severe incapacity was associated with higher FII titres (52). The group of Kim, Hyoun Ah pointed out that the neutrophil-to-lymphocyte ratio (NLR) can be used as a diagnostic tool and predictor of AOSD recurrence (53). Serum CXCL10, CXCL13 (54) and S100A12 (55) levels can be used as clinical markers to assess the disease activity of AOSD. Chen, D.Y’s team found that there is a fine-tuned mechanism between inflammatory and anti-inflammatory factors in AOSD (56), and IL-18 is an important predictor of active AOSD (57). In addition, galectin-3 (58), C-Type Lectin Domain Family 5-Member A (59), B19-NS1 (60), microRNA-134 (61), and the NLRP3 inflammasome (62) are involved in the pathogenesis of AOSD. Since 2018, the core research team formed by Wang, Zhihong, et al. from Shanghai Jiao Tong University has performed research on biomarkers of AOSD (63) and illustrated the pathogenesis, diagnosis, treatment and nursing of AOSD through clinical case data (64, 65). Their case study pointed out that neutrophils-derived lipocalin-2 can serve as an effective biomarker to identify AOSD with systemic inflammation (66).

The research field of AOSD can be divided into three phases in terms of time (**Figure 6** and **Table 7**) to better determine the research hotspots in different periods and the obvious change in research direction (67). In the early stage of research (1921–1997), researchers paid more attention to the therapeutic medication of disease. JACQUES M. G. W. WOUTERS et al.’s research shows that glucocorticoids can effectively relieve systemic and/or joint symptoms (68). Aydintuǧ, A. Olcay et al. showed that low-dose methotrexate (MTX) can treat AOSD and reduce the dosage of steroids (69). Researchers began to explore the pathological mechanism of disease and biomarkers related to disease in the middle of the research (1998-2011). Chen, Der Yuan’s research showed that Th1 cytokines may promote the pathogenesis of AOSD (70). In the recent stage (2012-2021), the complications of the disease became a hot topic for researchers. As seen, the focus of research has gradually changed from symptoms and treatment of AOSD to the complications and prognosis of diseases. Research on complications and new pathogenesis will become a research trend and hotspot in the future. However, slightly different from our research results, the research results of Xue Zhang et al. show that the research focus in the recent 10 years has mainly been on the treatment of AOSD and its serious complications (46). This difference may be mainly caused by the different literature source databases selected, different data analysis software used, and different literature data time spans included.

Combining the keyword frequency and keyword clustering analysis, the research hotspots and frontier are as follows (**Table 6** and **Figure 5**):

### #0 joint and #9 electrocardiography: affected positions of AOSD

Joints are the commonly affected areas of the AOSD and can occur singly or in combination. Joint problems are difficult to distinguish from other diseases, especially at the early stage of the disease (71). Common affected joints include the knee (43.9-82%), wrist (31.7-73%), ankle (31.7-55%), proximal interphalangeal (21.4-47%), metacarpophalangeal (21.4-46.3%) and other parts (72, 73). The heart is also one of the affected sites of AOSD, usually presenting as pericarditis (37%) (19), cardiac tamponade and myocarditis (74). Heart failure is the most serious manifestation of cardiac involvement, which may lead to death (75). Electrocardiogram (ECG) is a relatively simple and main method for diagnosing and monitoring cardiac involvement (76), and it is widely used in clinical practice. ECG can preliminarily judge whether patients have heart involvement and the degree of involvement to better help improve the follow-up examination and treatment, which are useful noninvasive diagnostic tools (74).

### #1 differential diagnosis and #6 arthritis rheumatoid: the differential diagnosis of AOSD

The diagnosis of AOSD primarily depends on the identification of other diseases. Rheumatoid arthritis is a common disease of the rheumatic immune system, with the main symptoms affecting the joints (77). AOSD is mainly distinguished from rheumatoid arthritis by the negative laboratory results of anti-citrullinated peptids autoantibodies and rheumatoid factor (16, 78). Other diseases that need to be identified mainly include Polyarteritis nodosa or other vasculitis, Polymyositis and systemic lupus (22).

### #2 prednisolone; #3 methotrexate; #5 drug therapy and #12 clotrimazole: drug therapy of AOSD

Treatment of AOSD begins with NSAIDs, but the response rate to these drugs is only 20–25% (79). Steroids are characterized by their ability to improve systemic manifestations in the acute phase, and they have a good therapeutic effect with a response rate of 76–95% (26), making them the first-line therapeutic drug in clinical practice at the present stage. However, about 45% of patients have steroid dependence and need DMARDs such as MTX to control the dose of steroids (80). At present, MTX is widely used in clinics and has good tolerance (28), about 70% of patients recover completely after MTX treatment (81). In addition, the treatment of prednisone refractory diseases with intravenous pulse methylprednisolone has been verified in some cases (82). The response rate of 60-80% can also be achieved by oral administration of glucocorticoids, such as prednisolone (71). Clotrimazole is an anti-fungal drug (83) that might produce an anti-inflammatory effect by stimulating the adrenal glands, which has been confirmed to be able to improve the symptoms of rheumatoid arthritis (84) and is a potential therapeutic drug for autoimmune diseases (85). Currently, many experiments have proven the feasibility of targeted biologic therapies (34). For example, targeted therapy is carried out by using drugs such as Tocilizumab (86), anti-human IL6 monoclonal antibody (87) and Anti-TNF-α (88).

### #4 macrophage activation syndrome; #8 complication; #11 prognosis; #13 leukemia: complication and prognosis of AOSD

MAS is one of the most common complications of AOSD, with an incidence rate of over 10% (89). The main symptoms include persistent fever, methemoglobinemia, pancytopenia and hepatic dysfunction (90). MAS is a potentially fatal inflammatory disease that lacks targeted therapy and relies primarily on combination immunosuppressive agents to relieve symptoms (91). Patients with hematopoietic malignancies, such as leukemia, may present with systemic symptoms consistent with AOSD (92). Therefore, to exclude hematological malignancies, the researchers suggest bone marrow aspiration and lymph node biopsy (93). In addition, a case reported that a patient was diagnosed with chronic myeloid leukemia two years after being diagnosed with AOSD, which also suggests the possibility of the development of complications (94). The prognosis of AOSD mainly depends on the severity of visceral involvement and the degree of joint erosion (7). Studies have shown that early diagnosis can improve prognosis, arthritis at diagnosis is an important predictor of disease chronicity, and high fever is a good predictor of systemic disease course (28). Few studies have discussed the prognosis of AOSD alone, which is mainly due to the heterogeneity of the disease (7).

### #7 arthritis, rheumatoid/pathology; #10 lipoprotein; #14 ferritin: the pathological mechanism of AOSD

Studies have shown that the chronic inflammatory response and joint destruction symptoms of RA are caused by genetic, environmental and immunological factors (95). Lipoprotein (a) is related to inflammatory reactions, and its monocytes can increase the production of pro-inflammatory cytokines after stimulation (96). Therefore, the increase in lipoprotein(a) levels in AOSD patients may also reflect a high inflammatory state (97). One of the clinical features of AOSD is a significant increase in serum ferritin levels (16). Ferritin is a pro-inflammatory mediator that can induce the expression of inflammatory molecules (98). High levels of ferritin are not only the product of inflammation but also contribute to the development of cytokine storms (99), which may lead to diseases (100). Cytokines such as IL-1β, IL-18, TNF, IFN-γ, and IL-6, which are all involved in the pathogenesis of AOSD, regulate the synthesis of ferritin (78). This finding indicates that ferritin may not only be a biomarker of AOSD but also be involved in the pathogenesis process of AOSD.

## Strength and limitations

To the best of our knowledge, little research systematically and comprehensively discusses the research progress and changing trends in the AOSD field. Therefore, to eliminate this limitation, we used CiteSpace software to visualize information such as authors, journals, and keywords. This study is the first time to analyze the literature in the field of AOSD in the past 100 years. By retrieving the Scopus database with a large amount of data in the field of AOSD, almost all the original studies in this field were included, and the history, current situation and trend analysis of the field of AOSD were conducted. On the one hand, this research provides valuable information for scholars in this field, which helps them understand the development process of AOSD and master the hot topics at the forefront; on the other hand, it also provides new research perspectives and ideas for exploring the development direction of AOSD.

However, this research inevitably has limitations that need to be solved in the future. Due to the continuous updating of the database and the limited analysis year span of CiteSpace software, only the articles from 1921 to November 30, 2021, were selected for this research, and articles published after that were not included in this research. Therefore, there will be discrepancies between bibliometric analysis and actual publication. Restricted by the capabilities of the analysis software, only articles in the Scopus core database are included in this research, which may result in potentially incomplete analytical data. Due to the limitation of CiteSpace software, a lack of unified parameter setting standards, data loss and partial data overlap will inevitably occur in the process of software clustering, which will also lead to the deviation of analysis results. In addition, according to the general process of bibliometrics research, this study adopts an accurate literature search strategy to ensure the correlation between downloaded literature and research topics and uses Cite Space software to remove duplicate literature. However, it is still possible to have subtle errors due to the database or software issues, which are slightly insufficient in artificial induction.

## Conclusions

The number of AOSD-related articles is generally on the rise. Developed countries have more research achievements and closer cooperation among countries and lack cooperation with other countries. The diagnosis and treatment of AOSD have always been the focus of researchers’ attention. In the future, the complications and new pathogenesis of AOSD will become research trends and hotspots.

## Data availability statement

The original contributions presented in the study are included in the article/supplementary material, Further inquiries can be directed to the corresponding author.

## Author contributions

AQ wrote the first draft of the manuscript. JS, CG wrote sections of the manuscript. CL contribute to the search strategy of the study. All authors contributed to manuscript revision, read, and approved the submitted version

## Conflict of interest

The authors declare that the research was conducted in the absence of any commercial or financial relationships that could be construed as a potential conflict of interest.

## Publisher’s note

All claims expressed in this article are solely those of the authors and do not necessarily represent those of their affiliated organizations, or those of the publisher, the editors and the reviewers. Any product that may be evaluated in this article, or claim that may be made by its manufacturer, is not guaranteed or endorsed by the publisher.
